# The Fitness Cost of Antibiotic Resistance in *Streptococcus pneumoniae*: Insight from the Field

**DOI:** 10.1371/journal.pone.0029407

**Published:** 2012-01-17

**Authors:** M. Cyrus Maher, Wondu Alemayehu, Takele Lakew, Bruce D. Gaynor, Sara Haug, Vicky Cevallos, Jeremy D. Keenan, Thomas M. Lietman, Travis C. Porco

**Affiliations:** 1 Department of Epidemiology & Biostatistics, University of California San Francisco, San Francisco, California, United States of America; 2 ORBIS International, New York, New York, United States of America; 3 F.I. Proctor Foundation, University of California San Francisco, San Francisco, California, United States of America; 4 Department of Ophthalmology, University of California San Francisco, San Francisco, California, United States of America; 5 Institute for Global Health, University of California San Francisco, San Francisco, California, United States of America; Centers for Disease Control & Prevention, United States of America

## Abstract

**Background:**

Laboratory studies have suggested that antibiotic resistance may result in decreased fitness in the bacteria that harbor it. Observational studies have supported this, but due to ethical and practical considerations, it is rare to have experimental control over antibiotic prescription rates.

**Methods and Findings:**

We analyze data from a 54-month longitudinal trial that monitored pneumococcal drug resistance during and after biannual mass distribution of azithromycin for the elimination of the blinding eye disease, trachoma. Prescription of azithromycin and antibiotics that can create cross-resistance to it is rare in this part of the world. As a result, we were able to follow trends in resistance with minimal influence from unmeasured antibiotic use. Using these data, we fit a probabilistic disease transmission model that included two resistant strains, corresponding to the two dominant modes of resistance to macrolide antibiotics. We estimated the relative fitness of these two strains to be 0.86 (95% CI 0.80 to 0.90), and 0.88 (95% CI 0.82 to 0.93), relative to antibiotic-sensitive strains. We then used these estimates to predict that, within 5 years of the last antibiotic treatment, there would be a 95% chance of elimination of macrolide resistance by intra-species competition alone.

**Conclusions:**

Although it is quite possible that the fitness cost of macrolide resistance is sufficient to ensure its eventual elimination in the absence of antibiotic selection, this process takes time, and prevention is likely the best policy in the fight against resistance.

## Introduction

*Streptococcus pneumoniae* is the leading cause of serious illness in children and adults worldwide [1]. Despite the availability of a vaccine to combat the disease, rates of colonization remain high, as vaccine-induced immunity often results in replacement of targeted strains as other pneumococcal serotypes fill the newly opened ecological niche [2]. This antigenic diversity renders eradication nearly impossible, emphasizing the importance of antibiotic treatment for control of invasive pneumococcal disease, and illuminating the necessity for understanding and predicting long-term trends in resistance.

The diversity and adaptability of *S. pneumoniae* is facilitated in large part by active DNA import and extensive genomic repeats that greatly increase the likelihood of intra- and interspecific homologous recombination [3]. Considering its high genomic plasticity, it is not surprising that substantial levels of drug resistance are observed in this organism in response to antibiotic treatment [4]. Yet, recent research has suggested that antibiotic resistance may come at a cost to bacteria harboring the trait, limiting fitness in the absence of antimicrobial drug selection [5].

Competition studies both *in vitro* [6], [7], [8] and in animal models [9], [10], [11] have found evidence for a competitive disadvantage from drug resistance in a range of bacterial species. At the epidemiologic level, several observational studies have demonstrated that lower antibiotic prescription rates are associated with reduced antibiotic resistance [12], [13]. Others have used correlations between resistance and prevalence to estimate fitness costs, either directly [14], [15] or by fitting a mathematical transmission model [16]. To our knowledge, however, no prior study has observed the development of antibiotic resistance upon introduction of a previously unused antibiotic class, and followed the decay of that resistance after antibiotic pressure is removed.

## Results

### Longitudinal trial data

**Figure 1:**
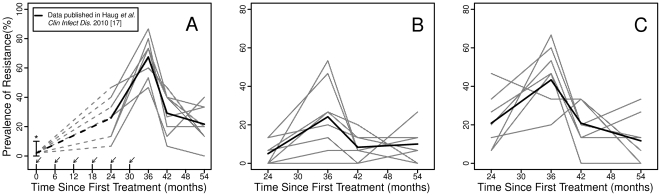
The prevalence of pneumococcal azithromycin resistance by village in the Ethiopian trachoma elimination study. **A**.) The overall prevalence of azithromycin resistance. Dotted lines denote extrapolation to estimated baseline levels of resistance based on a prevalence of resistance of 0.8% in control villages. Uncertainty in this estimate is represented with an error bar, and distinguished from observed data with an asterisk. Arrows mark times at which community-wide antibiotic treatment took place. B.) The prevalence of ermB-mediated azithromycin resistance C.) The prevalence of mefA/E-mediated azithromycin resistance.

Presented in Figure 1A is the village-level prevalence of azithromycin-resistant strains during the course of the study, with average prevalence superimposed. These average values have been presented previously by our group [17]. Briefly, average macrolide resistance rose from 28.2% after 4 treatments to 76.8% after the 6th treatment. Resistance then declined sharply to 30.6% at 12 months after the last antibiotic treatment, and finally to 20.8% one year after that.

To estimate baseline antibiotic resistance, we analyzed baseline data from eight of the control villages that were within a 5 mile radius of the intervention sites. Laboratory testing found only one resistant case among 112 pneumococcal isolates, a rate of approximately 0.9%. Combined with survey data showing that macrolides constituted approximately 0.5% of the antibiotic doses dispensed locally (B. Ayele, unpublished data), it is likely that baseline rates of azithromycin resistance were comparably low in intervention villages. For illustrative purposes, this assumption is included in Figure 1A with an error bar to represent its uncertainty, and an asterisk to distinguish it from directly observed data.

Figures 1B and 1C, respectively, show the proportion of azithromycin resistance due to the *mef*A/E and *erm*B phenotypes. We observed that pneumococcal strains harboring the *mef*A/E gene constituted a larger share of azithromycin-resistant samples than strains with the *erm*B phenotype. In addition, we observed that from month 42 to month 54, the overall decrease in azithromycin resistance was mostly due to the decrease in prevalence of *mef*A/E strains. In fact, in one village, we saw the measured prevalence of *erm*B strains increase from 0% to over 20% in one year. This suggests that reintroduction events played a role in long-term transmission dynamics, and in future studies, should be considered when feasible.

### Fitness cost estimation

Our model calculated the fitness of the *mef*A/E phenotype to be 0.86 (95% CI 0.80 to 0.90) relative to the azithromycin-sensitive strain, and similarly to be 0.88 (95% CI 0.82 to 0.93) for the *erm*B phenotype (log likelihood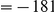). Table 1 demonstrates the sensitivity of these estimates to model inputs. Relative fitness estimates were insensitive to nearly all fixed parameters, with the exception of the duration of infection (1/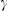) and the antibiotic efficacy against drug sensitive strains. In both cases, however, relative fitness estimates remained significantly below one across the range of input values tested.

**Table 1:** Parameter ranges tested during univariate sensitivity analysis, and their influence on calculated relative fitness.

Sensitivity analysis					
Parameter	Baseline value	Range tested	Relative Fitness		Log-likelihood
			*mef*A/E	*erm*B	
Duration of infection (1/ 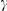 ) in weeks		8	.74 [.69–.80]	.78 [.71–.84]	−162
		6	.79 [.74–.85]	.82 [.76–.88]	−166
		2	.93 [.89–.97]	.94 [.90–.98]	−250
Sensitive strain transmission rate constant ( 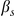 )	3.25	2.5	.86 [.81–.90]	.88 [.83–.93]	−184
		4	.85 [.81–.91]	.87 [.83–.93]	−184
Antibiotic coverage*efficacy for drug sensitive strains (%)	88	70	.90 [.85–.95]	.92 [.87–.97]	−178
		99	.76 [.72–.81]	.79 [.74–.84]	−269
Antibiotic coverage*efficacy for *mef*A/E strains (%)	5	1	.85 [.80–.90]	.88 [.82–.93]	−182
		10	.86 [.81–.91]	.87 [.82–.93]	−182
Antibiotic coverage*efficacy for *erm*B strains (%)	5	1	.86 [.81–.90]	.87 [.82–.92]	−182
		10	.86 [.81–.90]	.88 [.83–.93]	−183

Units for 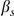 are per infective, per susceptible, per week.

### Probability of Strain Survival

**Figure 2:**
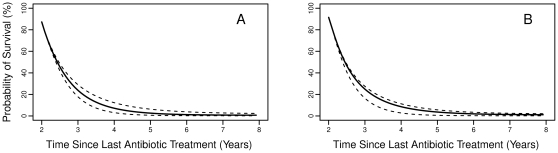
The probability of survival versus time under the basecase model for A.) the mefA/E strain and B.) the ermB strain. Upper and lower dashed lines, respectively, correspond to the probability of survival under the upper and lower bounds of the relative fitness estimate.

To examine the effect of fitness cost on predicted long-term trends in resistance, we performed simulations extrapolating the probability of survival for each azithromycin-resistant strain, out to 8 years after the last treatment. Figure 2 demonstrates that, under the basecase point estimate for the relative fitness, we would expect less than a 5% chance of survival for both drug resistant strains 5 years after the last antibiotic administration. Using the less optimistic upper bound for this estimate, we find the probability of survival at this time point to be only slightly higher: 6% for the *mef*A/E strain, and 5.4% for the *erm*B strain.

## Discussion

Previous research has demonstrated that antibiotic resistance often comes with a fitness cost, the magnitude of which may depend on growth conditions, mechanism of resistance, genetic background, and the presence of compensatory mutations [5]. *In vitro* and animal model experiments are capable of analyzing the influence of these variables, but in humans, even quantifying an average fitness cost of drug resistance is made difficult by the increased complexity and decreased control introduced by working with field data. As a result, most epidemiological studies interested in the fitness cost of drug resistance in *S. pneumoniae* have looked at correlations between naturally occurring changes in prescription rates and the prevalence of corresponding antibiotic resistances [14], [18]. Although this has demonstrated a significant relationship between the two factors, lack of an underlying transmission model and relatively small changes in antibiotic consumption have hindered the estimation of relative fitness values.

In this study, we have used data gathered as part of a longitudinal trachoma elimination study to examine the fitness cost of macrolide resistance in *S. pneumoniae*. Observed macrolide resistance rates rose from 28.2% following four rounds of biannual mass antibiotic distribution, to 76.8% after two subsequent rounds, followed by a drop to 20.6% twenty-four months after the conclusion of the program. Fitting a mathematical model to these data, we estimated the relative fitness for strains harboring two different mechanisms of macrolide resistance, and showed how these estimates may depend on assumed transmission parameters and antibiotic effectiveness. Calculated fitness costs increased significantly with higher antibiotic efficacy against drug-sensitive strains, and longer durations of infection (1/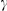). These results agree with theory. Respectively, a larger available ecological niche, or fewer infection cycles, would both be expected to magnify the competitive disadvantage of drug resistance given identical data.

The clinical significance of these differing fitness costs can be measured, in part, by antibiotic-resistant strains' probability of survival versus time. Our model predicts that, in the base scenario, it may take up to 5 years for macrolide resistant strains of *S. pneumoniae* to reach high probabilities of elimination by competition alone. In fact, this number may be an underestimate of the true value, as our model does not allow for the stable coexistence of pneumococcal strains. Previous modeling studies have shown that coexistence can be explained by simultaneous carriage of multiple strains [19], a phenomenon we were unable to model due to a lack of data on superinfection. However, the decreased complexity of the model allowed us to follow the probability of all community-level infection states over time, better capturing the stochastic effects that are important in small populations.

It should also be noted that due to bias inherent in our phenotyping method, any individual strains with both mechanisms of resistance would be classified as *erm*B by our protocol. A study from a different area of Ethiopia observed these strains to constitute 6.6% of resistant samples after 4 rounds of azithromycin treatment [20]. This misclassification would most likely increase the estimated fitness cost for the *erm*B strain, although low prevalence of *mef*A/E+, *erm*B+ strains would attenuate this effect.

In addition, our model does not allow for strain reintroduction, or for the amelioration of fitness costs by compensatory mutation, an adaptation that has been observed to develop quickly in other organisms [5]. The rate of compensatory mutation is only one part of the picture, however. In pneumococcal, both mechanisms of macrolide resistance are mediated by entire genes rather than single nucleotide polymorphisms (SNPs). As a result, the genetic target for mutations that attenuate or even eliminate resistance is comparatively much larger, consequently making these events far more likely. In other words, in the absence of antibiotic selection against pneumococcus, we would expect to see two opposing effects not captured in our model. Mutation could reduce antibiotic resistance, as well as the fitness cost associated with it.

Notwithstanding uncertainty in the long-term prediction of resistance rates, the existence of an epidemiologically verified fitness cost of macrolide resistance in *S. pneumoniae* suggests that intraspecific competition may be exploited to at least partially reclaim antibiotic efficacy after the development of high levels of resistance. In practice, however, this process may be highly variable and take years even under the most optimistic scenarios. Although it is quite possible that the fitness cost of macrolide resistance is sufficient to ensure its eventual elimination in the absence of antibiotic selection, this process takes time, and prevention is likely the best policy in the fight against resistance.

## Methods

### The Trachoma Elimination Follow-up Study

The Trachoma Elimination Follow-up (TEF) study [21] is a longitudinal cohort study conducted in the Gurage Zone in Ethiopia, in which 40 villages were chosen from the Gurage Zone in Ethiopia, assessed for ocular chlamydial infection, randomized to one of 5 treatment protocols, and monitored over a 54 month period. Individuals aged 1 year or older were offered directly observed treatment with single-dose oral azithromycin (1 g in adults; 20 mg/kg in children). Pregnant women or those allergic to macrolides were ordered a six-week course of topical 1% tetracycline ointment to be applied twice daily to both eyes (not directly observed).

A subset of 8 villages was selected randomly selected from the biannual treatment arm for a more detailed pneumococcal antibiotic resistance investigation. During the follow-up, we collected pneumococcal samples from fifteen randomly chosen one-to-five year olds in each village (a total of 120 children were studied at each time point). Nasopharyngeal swabs were taken at 24, 36, 42, and 54 months. To estimate pneumococcal prevalence prior to treatment, 240 children (also aged 1–5 years old) were randomly selected and sampled from sixteen nearby villages prior to the enrollment of the villages in the treatment program.

Verbal consent was obtained from the guardians of all children participating in the study. Written consent was not sought due to the low literacy rate in the area. Verbal consent was approved by both the University of California, San Francisco Institutional Review Board (IRB), and the Ethiopian IRB (The Ethiopian Science and Technology Commission). Consent was documented on the encounter form by a check mark. Approval for the study as a whole was also obtained from the UCSF IRB and the Ethiopian IRB. The study was carried out in accordance with the Declaration of Helsinki.

### Determining Strain Assignments

Fifteen samples were collected from each village at each time point, tested for resistance to a variety of antibiotics, and then classified as (1) azithromycin-sensitive (2) azithromycin-resistant, with *mef*A/E phenotype or (3) azithromycin-resistant, with *erm*B phenotype. All samples with mean inhibitory concentrations (MIC) of azithromycin less than 1 mg/L where categorized as drug-sensitive. For samples with MICs over this threshold, individual-level antibiotic resistance profiles were used to determine the mechanism of macrolide resistance.

This inference was possible because more than 97% of pneumococcal resistance to macrolides is mediated by one of two genotypes [22]. Azithromycin resistance arising from the *erm*B genotype also gives rise to resistance to lincosamides, such as clindamycin [23]. In contrast, *mef*A/E offers no such cross-protection, allowing for discernment between the two mechanisms by examining whether we also observe clindamycin resistance [24], [25].

It is important to note here that our phenotyping method would classify *mef*A/E+, *erm*B+ strains as being *erm*B+ only. Data from a different area of Ethiopia showed that *mef*A/E+, *erm*B+ strains constituted 6.6% of resistant strains after four rounds of azithromycin treatment [20]. It is likely that strains carrying both resistance alleles would have increased antibiotic resistance, but also lowered fitness. Although our model is insensitive to the assumption of antibiotic resistance rates for drug-resistant strains (see Table 1), it is possible that this misclassification could amplify the estimated fitness cost of *erm*B strains.

### Constructing the model

We employed a dynamic SIS (susceptible-infective-susceptible) model including *mef*A/E, *erm*B, and antibiotic-sensitive pneumococcal strains, so that within each village, there was a given number of individuals who were uninfected, infected with drug susceptible strains, infected by *mef*A/E strains, and infected by *erm*B strains. Between mass treatments, these numbers changed as individuals recovered or became newly infected. During a mass treatment, each individual's probability of cure was the product of the chance of treatment and the chance of cure given the individual's infection status. All equations relevant to this model are included in Supporting Information S1.

The model focused on children between the ages of one and five, as this group has the highest rates of acquisition, and new cases within the age group appear to occur primarily as the result of interaction with fellow members [26], [27]. Based on the distances between villages and the lack of motorized transportation in the area, transmission between villages was assumed to be negligible.

A mixed discrete-continuous time system was used to model this process, with ordinary differential equations (Equation A2) governing the probability that a village has given numbers of individuals of each status between mass treatments. The probability of a given village state following a mass treatment was the sum of the products of two probabilities: the probability of being in a given state, and the probability of transitioning from that state to the state of interest (Equation A2).

We initialized the system of equations using the first set of available village-specific pneumococcal sample results (taken at month 24 after the beginning of the study). Assuming *a priori* no preference for any given number of individuals infected with each of the three possible strains, we used the observed sampling results to compute a posterior probability distribution for the number of infected individuals in each village (see Equation A1). This posterior distribution was used as the initial condition for data-fitting simulations. Similarly, extrapolation of the probability of extinction versus time was achieved by initializing the maximum likelihood model with our observations at month 54.

In both cases, all strains were assumed to have the same rate of recovery (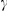), which was extracted from the literature [28]. It should be noted that younger children may exhibit a slower average rate of recovery than our baseline value. As we see in the sensitivity analysis (Table 1), the resulting longer durations of infection imply larger proportional fitness costs, since the reduction in observed prevalence must take place over fewer generations of transmission. The infection rate constant for the antibiotic-sensitive strain, 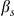, was calculated from the equation: equilibrium prevalence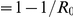, where 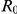 is the basic reproduction number. Infection rate constants for the antibiotic-resistant strains were allowed to vary during the optimization. In several cases, values examined in the sensitivity analysis improved the likelihood of the model. For the basecase, we chose to keep the parameters that we judged to be the closest match to values from the literature.

For fitness cost estimation, cure rate parameters were also selected for each strain to probabilistically model treatment at months 24 and 30 (Equation A2). These values were chosen based on survey data (B. Ayele. unpublished data), and pneumococcus-specific pharmacodynamics simulations [29]. Comparing these data with observed MIC values allowed us to calculate an expected cure rate for *mef*A/E of 5%. The cure rate for *erm*B strains was estimated to be near-zero by this method, but was also set to 5% to avoid introducing an *ab initio* difference between the two strains. The cure rate was estimated to be 88% for the antibiotic-sensitive strain, by extrapolating estimated treatment efficacies of around 93% from studies that used higher doses of azithromycin, but in populations that presumably harbored resistance [30], [31].

Between treatments, we integrated the ordinary differential equations for the probability of each village state (equation A2) using the GNU Scientific Library [32]. From these probabilities, we used Equation A4 to calculate the likelihood function for observing the village-level strain type data over time for each village (taking the number of children per village to be 50, consistent with survey results). In short, for each village we summed over the probability of each 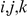 infection state multiplied by the probability of our observations at that time point, given that state. Assuming the villages to be independent, we then multiplied these village-level probabilities together to get an overall probability of the data at that time point given the parameters. These time point probabilities were then multiplied together to get the overall likelihood of the data given the parameters.

We optimized this likelihood with respect to the *mef*A/E and *erm*B transmission rate constants, 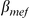 and 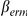, using the optim function in R (http://www.r-project.org). All other parameters were held fixed, and subsequently examined by univariate sensitivity analysis. Asymptotic standard errors for these estimates were found using the observed Fisher information. Finally, the upper bound, lower bound, and point estimates for the relative fitness values of the *mef*A/E and *erm*B strains were used to extrapolate each strain's probability of survival versus time.

## Supporting Information

Supporting Information S1 This file describes the equations relevant to the construction of our model.
(PDF)
