# Supplementary material for: The Fitness Cost of Antibiotic Resistance in Streptococcus pneumoniae: Insight from the Field
Source: PLoS One. 2012 Jan 17;7(1):e29407. doi: 10.1371/journal.pone.0029407 (PMC3260144; doi:10.1371/journal.pone.0029407)
Supplement: Supporting Information S1 — This file describes the equations relevant to the construction of our model. (PDF) [file pone.0029407.s001.pdf]

Supporting Information

In each village, we let X be the number of 1-5 year-old children uninfected with pneumococcus, I be the number infected with *erm*B, J be the number infected with *mef* A/E, and K be the number infected with azithromycin-sensitive strains. We then modeled $P(X(t) = x, I(t) = i, J(t) = j, K(t) = k)$, with $x+i+j+k = N$, as a continuous-time Markov process. We represent this joint probability as $p_{i,j,k}(t)$. Observed values are denoted with the subscript *obs*.

Calculating Initial Conditions

The posterior distribution for each village is given by the following formula:

$$p_{i,j,k|i_{obs},j_{obs},k_{obs}}(t_0) = \frac{\binom{i}{i_{obs}}\binom{j}{j_{obs}}\binom{k}{k_{obs}}\binom{x}{x_{obs}}}{\binom{50}{15}} p_{i,j,k}(t_{prior}) \Bigg/ \sum_{i,j,k} \frac{\binom{i}{i_{obs}}\binom{j}{j_{obs}}\binom{k}{k_{obs}}\binom{x}{x_{obs}}}{\binom{50}{15}} p_{i,j,k}(t_{prior})$$

where 50 corresponds to the number of children per village, and 15 is the number of nasopharyngeal swabs collected in each village at each time point.

Modeling Treatment

Let E be the treatment efficacy against *erm*B strains, M be the treatment efficacy against *mef* A/E strains, and S be the treatment efficacy against antibiotic sensitive pneumococcal strains. We can then model treatment as independent binomial draws, as shown in the following equation:

$$\begin{aligned} &p_{i,j,k}(t+1) = \dots \\ &p_{i,j,k}(t)\left(1 - \sum_{l=0}^{(i-1)}\sum_{m=0}^{(j-1)}\sum_{p=0}^{(k-1)} \binom{i}{i-l} E^{i-l}(1-E)^l \binom{j}{j-m} M^{j-m}(1-M)^m \binom{k}{k-p} S^{k-p}(1-S)^p\right) \\ &+ \sum_{x=i+1}^{(N-j-k)}\sum_{y=j+1}^{(N-x-k)}\sum_{z=k+1}^{(N-x-y)} p_{x,y,z}(t) \binom{x}{x-i} E^{x-i}(1-E)^i \binom{y}{y-j} M^{y-j}(1-M)^j \binom{z}{z-k} S^{z-k}(1-S)^k \end{aligned}$$

In other words, the probability of being in state $p_{i,j,k}$ at time t+1 is equal to the probability of being in that state at time t, times the probability of remaining in it, plus the total flow into that state from higher infection states.

Differential Equations

$$
\begin{aligned}
\frac{dp_{i,j,k}(t)}{dt} =& - p_{i,j,k}(t)\left((N-i-j-k)\left(\frac{i}{N}\beta_{mef} + \frac{j}{N}\beta_{erm} + \frac{k}{N}\beta_s\right) + i\gamma_{mef} + j\gamma_{erm} + k\gamma_s\right) \\
&+ p_{i-1,j,k}(t)\,(N-(i-1)-j-k)\left(\frac{i-1}{N}\right)\beta_{mef} \\
&+ p_{i,j-1,k}(t)(N-i-(j-1)-k)\left(\frac{j-1}{N}\right)\beta_{erm} \\
&+ p_{i,j,k-1}(t)(N-i-j-(k-1))\left(\frac{k-1}{N}\right)\beta_s \\
&+ p_{i+1,j,k}(t)(i+1)\gamma \\
&+ p_{i,j+1,k}(t)(j+1)\gamma \\
&+ p_{i,j,k+1}(t)(k+1)\gamma,
\end{aligned}
$$

where β_{mef}, β_{erm}, and β_s denote the transmission coefficients for each strain.

Calculating the Joint Likelihood

Let v be the index corresponding to each village, and let i,j,k, and x be the predicted number of children in each of the 4 groups under the model. Let i_{obs}, j_{obs}, k_{obs}, and x_{obs} be the number of children observed to be in each group by analysis of nasopharangeal swabs, and θ be the vector of model parameters. Then, the joint likelihood of the observations under the model can be calculated by:

$$
\mathcal{L}(\theta|data) = \prod_{v=1}^{8} \prod_{t\in(36,42,54)} \left(\sum_{i,j,k} p_{i,j,k|\theta}(v,t) * \mathcal{S}(v,t) \right)
$$

where $\mathcal{S}(v,t)$ is the probability of the observed samples, given i, j, and k:

$$
\mathcal{S}(v,t) = \frac{\binom{i}{i_{obs}(v,t)}\binom{j}{j_{obs}(v,t)}\binom{k}{k_{obs}(v,t)}\binom{x}{x_{obs}(v,t)}}{\binom{50}{15}}
$$
